# Chloroplast budding mediates β-carotene transport for early stage astaxanthin hyperaccumulation in microalgae

**DOI:** 10.1093/plphys/kiaf423

**Published:** 2025-10-13

**Authors:** Haiyan Ma, Zhaokun Wang, Feng Ge, Cheng-Cai Zhang

**Affiliations:** State Key Laboratory of Breeding Biotechnology and Sustainable Aquaculture, Institute of Hydrobiology, Chinese Academy of Sciences, Wuhan 430072, China; Institute of Hydrobiology, Chinese Academy of Sciences, Wuhan 430072, China; University of Chinese Academy of Sciences, Beijing 100049, China; State Key Laboratory of Breeding Biotechnology and Sustainable Aquaculture, Institute of Hydrobiology, Chinese Academy of Sciences, Wuhan 430072, China; Key Laboratory of Algal Biology, Institute of Hydrobiology, Chinese Academy of Sciences, Wuhan, Hubei 430072, China; Key Laboratory of Algal Biology, Institute of Hydrobiology, Chinese Academy of Sciences, Wuhan, Hubei 430072, China; Key Laboratory of Lake and Watershed Science for Water Security, Chinese Academy of Sciences, Nanjing 210008, China; Hubei Hongshan Laboratory, Wuhan 430070, China

## Abstract

β-Carotene, the precursor of the high-value carotenoid astaxanthin, is transported into the cytoplasm via chloroplast membrane budding.

Dear Editor,

Carotenoids, a diverse family of natural terpenoids, are ubiquitous in photosynthetic organisms (Rodriguez-Concepcion et al. 2018; Sun et al. 2022), playing crucial roles in light absorption, photoprotection, and pigmentation (Domonkos et al. 2013; Hashimoto et al. 2016). While plants store these pigments in specialized plastid-derived chromoplasts (Sun et al. 2022), microalgae predominantly accumulate them in lipid bodies either within or outside of the chloroplast (Pick et al. 2019). *Haematococcus pluvialis*, a green microalga native to shallow, temporary water bodies, is renowned for its ability to hyperaccumulate the potent antioxidant astaxanthin, making it a promising candidate for biotechnological applications (Han et al. 2013). In this study, we investigated the early phase of astaxanthin accumulation under nonstressful, baseline conditions, which not only initiate astaxanthin accumulation in *H. pluvialis* but also preserve cell motility. Our findings unveil a striking phenomenon: β-carotene/astaxanthin-rich globules originating from chloroplasts, which fluoresce distinctly under carotenoid excitation and are intimately associated with chloroplast membranes. We demonstrate the budding of β-carotene-enriched plastoglobuli (PG) from chloroplasts, suggesting an ancient mechanism akin to chromoplast biogenesis in land plants. Transcriptomic analysis revealed that 56 genes related to vesicle transport and membrane budding are upregulated under low-light conditions, with four of these genes being uniquely expressed in *H. pluvialis*. These findings provide insights into the molecular drivers behind the alga's hypercarotenogenic response and the origin of chromoplasts in eukaryotic photosynthetic organisms.

*H. pluvialis* cells exhibited an atypical brownish pigmentation during routine culture, differing from standard green vegetative cells or stress-induced red cysts. Algae were cultured at 22 °C with a light intensity of 10 *µ*mol m^−2^ s^−1^ under a 12h:12 h light:dark cycle in Basal medium (Kobayashi et al. 1991). Cultures were grown until reaching a density of 2 × 10^5^ cells ml^−1^. The algae were then subjected to continuous light at 25 *µ*mol m^−2^ s^−1^ (low light) at 22 °C. This condition was intended to examine the early phase of astaxanthin accumulation under nonstressful, baseline conditions, without the application of typical stress inducers such as high-light intensity, elevated temperature, salinity, or nutrient limitation, which are often employed in other studies to promote astaxanthin biosynthesis (Han et al. 2013). Sustained astaxanthin accumulation was achieved under these conditions without compromising cellular motility (Fig. 1A, Supplementary Fig. S1 and Movie S1). No cytoplasmic pigment was detected within 12 h, with chloroplasts dominating cell volume. By 24 h, Cy3-fluorescent structures (presumed astaxanthin complexes with autofluorescence at λ_ex_ = 540 nm/λ_em _= 600 nm) appeared adjacent to chloroplasts, suggesting a close spatial association. These red–orange structures progressively expanded, forming vesicles that segregated chloroplasts from cytoplasm by 48 h (Fig. 1B). At 72 h, intracellular pigment intensified and extracellular vesicles became apparent. Time-course analysis revealed progressive astaxanthin accumulation in *H. pluvialis*, reaching 0.02%, 0.17%, and 0.58% of dry weight at 24, 48 and 72 h, respectively (Supplementary Fig. S1), while the cells maintained motility at 72 h (Supplementary Movie S1).

**Figure 1.**
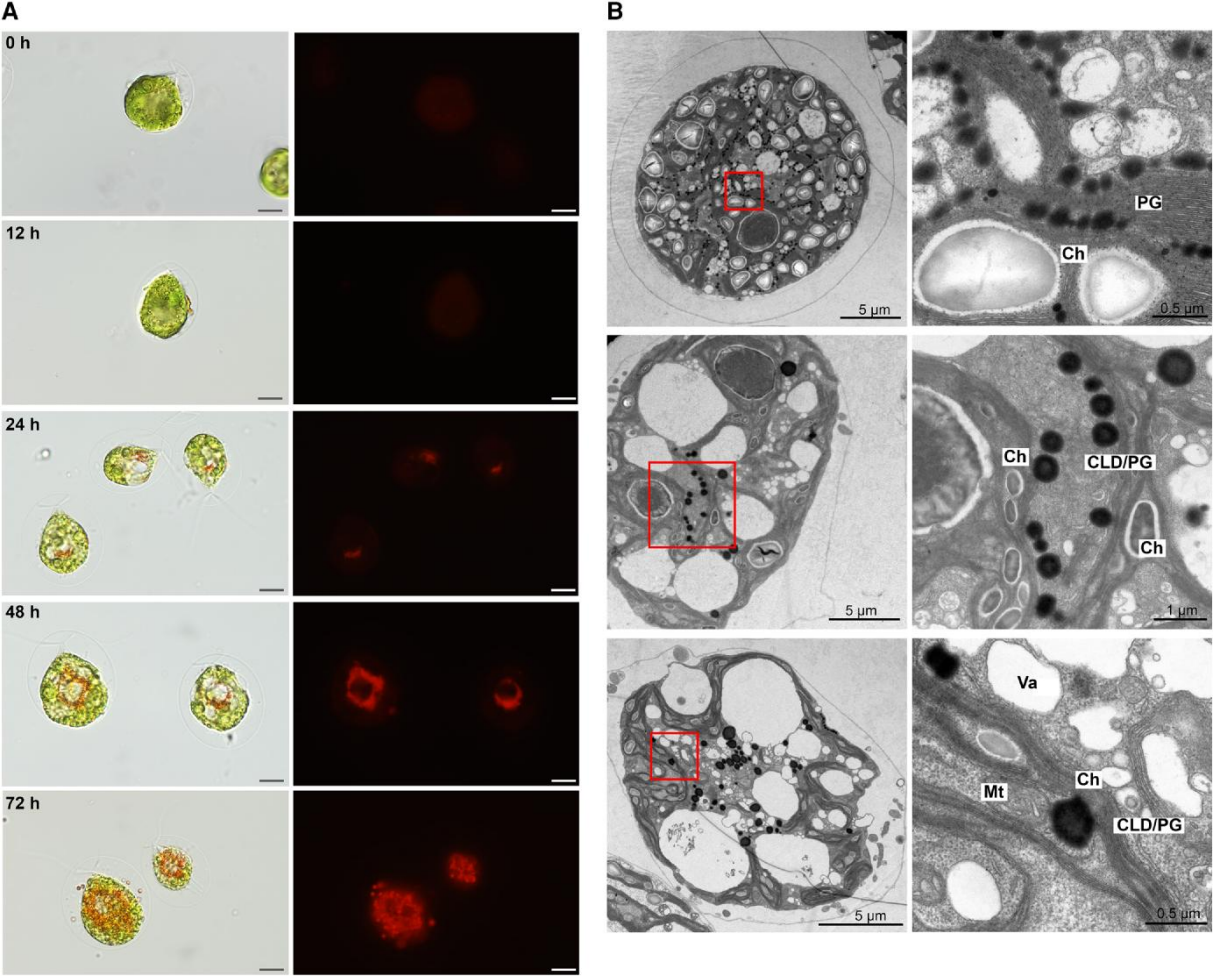
β-carotene transport dynamics in *H. pluvialis* NIES-144 under low-light induction (25 *µ*mol m⁻²s⁻¹ continuous illumination in Basal medium). **A)** In situ astaxanthin accumulation visualized by autofluorescence microscopy (Zeiss, Axio Imager A2). Detection channel: Cy3 (λ_ex_537.5–562.5 nm/λ_em_570–640 nm). Scale bar: 10 *µ*m. **B)** Ultrastructural analysis of PG dynamics: Chloroplast periphery accumulation of osmiophilic PGs (upper); CLDs containing translocated PGs (middle); PG budding from chloroplast membranes (lower). PG: plastoglobuli (osmiophilic globules completely embedded within chloroplasts). CLD: cytosolic lipid droplets (less osmiophilic than PGs and physically separate from chloroplasts). CLD/PG: budding PG (osmiophilic globules external to, but structurally connected to chloroplasts). Ch: chloroplast. Mt: mitochondria. Va: vacuole. The right panel in each row shows an enlarged view of the region indicated by the red boxes in the left panel.

To investigate carotenoid transport using confocal microscopy, specific inhibitors were employed: the β-carotene ketolase (BKT) inhibitor diphenylamine (DPA) to increase β-carotene accumulation by blocking its conversion to canthaxanthin (Harker and Young 1995), thereby facilitating transport visualization, and the DGAT inhibitor xanthohumol (Inokoshi et al. 2009) to assess lipid bodies' role in carotenoid trafficking by inhibiting triacylglycerol (TAG) accumulation. This approach revealed distinct carotenoid transport dynamics (Supplementary Fig. S2). In the control group, β-carotene was detected both inside and outside the chloroplast. In some regions, strong astaxanthin signals overlapped with β-carotene and colocalized with the chloroplast (indicated by cyan boxes in Supplementary Fig. S2), suggesting that certain β-carotene/astaxanthin-containing vesicles may be physically associated with chloroplast membranes. DPA treatment maintained cell integrity but reduced the vacuolar size and induced the formation of cytoplasmic β-carotene–rich lipid bodies. The astaxanthin signal was largely absent under these conditions, while the intracellular localization of chlorophyll remained unchanged. Conversely, DPA-xanthohumol cotreatment disrupted chloroplasts and extracellular matrices, trapping β-carotene in chloroplast foci while abolishing astaxanthin. High-performance liquid chromatography confirmed DPA reduced astaxanthin by 40% and increased β-carotene, with combined treatments eliminating astaxanthin (Supplementary Fig. S3). These results demonstrate β-carotene exits chloroplasts for cytosolic astaxanthin synthesis, initiated at chloroplast–cytosol interfaces under low light. Furthermore, lipid body formation is required for β-carotene transport from chloroplast.

Microscopic analysis revealed chloroplasts closely associated with astaxanthin-containing cytoplasmic lipid droplets (CLD). Intact chloroplasts isolated via hypertonic shock, membrane filtration, and Percoll gradient centrifugation (Supplementary Fig. S4A) showed a strong association with of astaxanthin-containing vesicles (likely also contain β-carotene) structurally linked to chloroplast surfaces (Supplementary Fig. S5), despite minor organelle contamination (Supplementary Fig. S4B).

Transmission electron microscopy (TEM) of low-light-induced cells demonstrated that osmiophilic plastoglobuli (PG), initially localized within chloroplasts, expanded and budded outward under stress (Fig. 1B, Supplementary Fig. S6). These PG exhibited higher electron density centrally during early budding and peripherally after cytosolic release. PG-mediated β-carotene transport was complemented by cytoplasmic vacuoles embedding budding PG for CLD delivery (Supplementary Fig. S7A). Large CLD near chloroplasts accumulated β-carotene at chloroplast–CLD interfaces, suggesting direct transfer (Supplementary Fig. S7B).

Transcriptomic analysis of low-light-induced *H. pluvialis* identified 6,588 differentially expressed genes (DEGs), including 126 linked to vesicular/vacuolar transport (56 upregulated, 70 downregulated) (Supplementary Fig. S8 and File S1). Unlike *H. pluvialis*, in land plants and halophilic green algae such as *Dunaliella bardawil* and *Dunaliella salina*, carotenoids, predominantly β-carotene, are stored in PG within plastid-derived organelles such as chloroplasts or chromoplasts (Davidi et al. 2015; Yuan et al. 2015; Sun et al. 2018; Pick et al. 2019). We proposed that DEGs related to vesicle or vacuolar transport, specifically those present in *H. pluvialis* but absent in *Dunaliella*, are likely involved in the β-carotene–rich PG budding and translocation from the chloroplast. In *Dunaliella*, which lacks BKT and shows no evidence of cytosolic carotenoid trafficking, β-carotene remains confined within chloroplast PG. Cross-species screening [e.g. *Chlamydomonas reinhardtii*, *Arabidopsis thaliana*, *Dunaliella* sp., tomato (*Solanum lycopersicum*), wild carrot (*Daucus carota*), and African marigold (*Tagetes erecta*)] prioritized seven upregulated candidates absent in β-carotene–accumulating *Dunaliella* sp. (Fig. 2A, Supplementary Table S1). Unigene23180, lacking homologs in tested species, showed progressive upregulation (1.5- to 5.8-fold) during induction. Three genes (20261, 24795, 5923) were unique to *C. reinhardtii*, with unigene5923 rising 52-fold by 72 h. Unigene23180, Unigene20261, Unigene5923, and Unigene20678 exhibited significantly upregulated expression at 12 or 24 h, or both time points under high-light stress conditions (200 *µ*mol m^−2^ s^−1^; Supplementary Fig. S9 and Supplementary Table S2). Although astaxanthin accumulation is enhanced under prolonged high-light conditions, the downregulation of these genes after 24 h suggests a potential shift in the β-carotene transport strategy. We propose that *H. pluvialis* may transition from vesicle-mediated transport to more direct pathways, such as a physical connection between the chloroplast and CLDs (see Supplementary Fig. S7B), which may serve as the primary route for sustained astaxanthin biosynthesis. Unigene20261 shows low sequence similarity (below 30% query coverage), indicating substantial divergence between *H. pluvialis* and *C. reinhardtii*. The homologs of unigenes 24795 and 5923 in *C. reinhardtii* suggest that these genes may be specific to green microalgae. Two others (20678, 17982) were absent only in *Dunaliella*. Divergent expression and absence of these genes in *Dunaliella* suggest the possible specialized roles in stress-induced β-carotene transport in *H. pluvialis*. In silico analysis of these DEGs identified conserved domains in YbbA, Vsp54, and Sec8 (Supplementary Table S1). Vsp54 and Sec8 are known vesicular transport components (TerBush and Novick 1995; Conibear and Stevens 2000). The remaining genes were annotated for vesicle-mediated transport via GO analysis. DeepLoc-2.1 predicted subcellular localization, membrane association type, and potential sorting signals: Unigene24795 (lysosome/vacuole; peripheral/soluble; signal peptide); Unigene5923, Unigene20678, Unigene17982 (cytoplasm; peripheral/soluble; none); Unigene23180 (cytoplasm, nucleus, membrane, or mitochondrion with low confidence; soluble; none).

**Figure 2.**
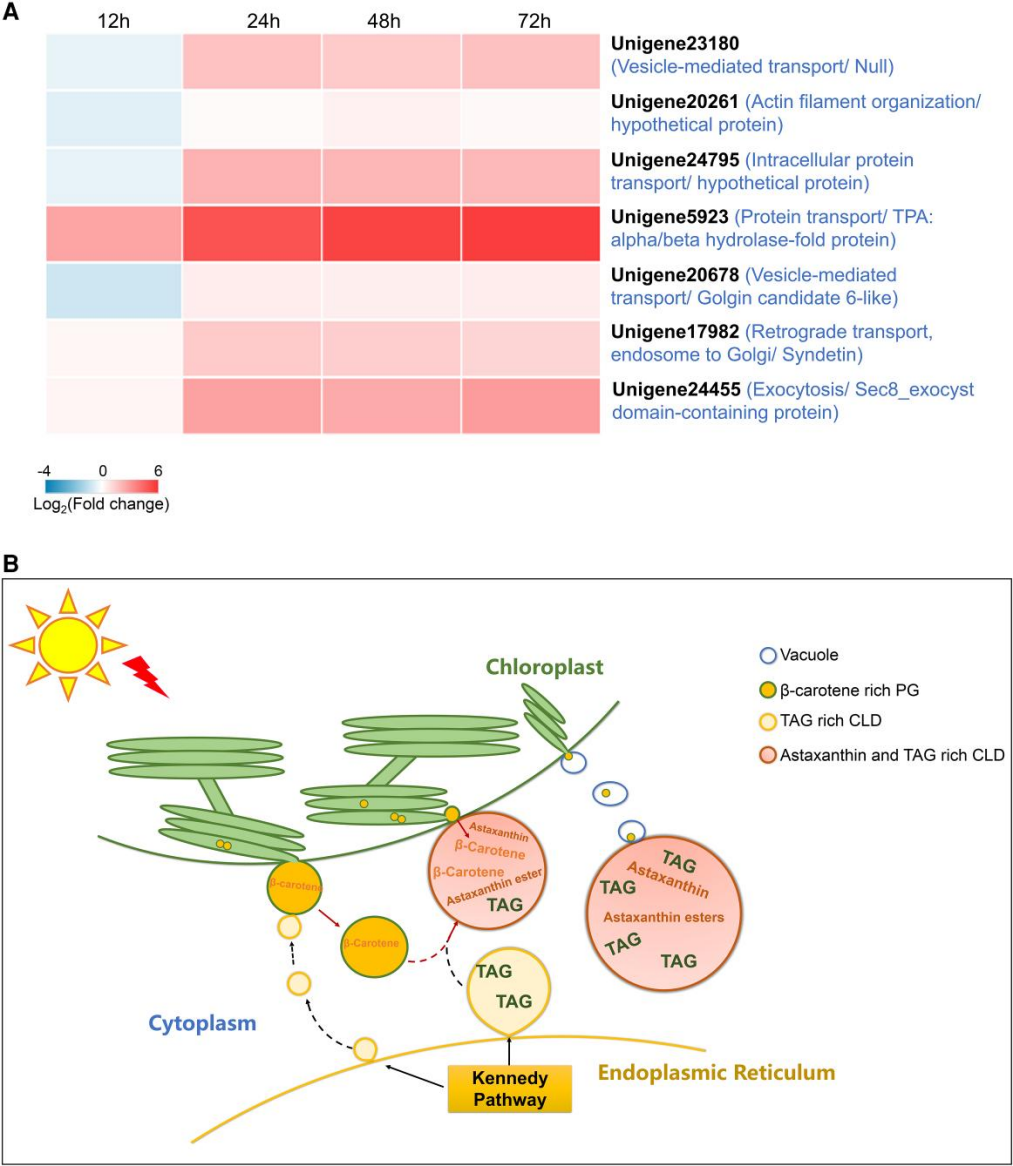
Proposed β-carotene trafficking mechanism in *H. pluvialis* NIES-144 chloroplasts under light stress and associated vesicular transport machinery. **A)** Transcriptional regulation of vesicular transport components identified in *H. pluvialis* but absent in *Dunaliella* species (*D. salina*, *D. bardawil*) during 72-h low-light induction (25 *µ*mol m^2^s^−1^). Heatmap displays Log_2_(fold change) of DEGs with Gene Ontology (GO) terms of ESCRT, multivesicular, vacuolar transport, clathrin, vesicle, budding, or SNAR. Differential gene expression analysis was performed comparing induction time points (12, 24, 48, and 72 h) against the 0 h baseline using DESeq2/DEGSeq, with significance thresholds set at |Fold Change| ≥ 2 and adjusted P-value (FDR) ≤ 0.001. Gene identifiers with functional descriptions are provided in parentheses. Hierarchical clustering was constructed of triplicate biological samples. **B)** Hypothesized transport pathway integrating microscopic and ultrastructural observations: PG biogenesis at chloroplast membranes; CLD formation via PG budding; vacuole-assisted PG translocation; membrane contact sites mediating CLD-PG exchange. TAG, triacylglycerol.

The hyperaccumulation of astaxanthin in CLD in *H. pluvialis* under stress conditions is analogous to carotenoid storage in chromoplasts of land plants. Chromoplasts, which accumulate carotenoids in colorful fruits, flowers, and vegetables (Egea et al. 2010; Li and Yuan 2013; Schweiggert and Carle 2017), serve dual roles similar to astaxanthin sequestration in CLD in *H. pluvialis*: (1) stabilizing carotenoid storage and (2) sustaining biosynthesis by preventing plastid membrane overload in plastids (Merzlyak and Solovchenko 2002; Li et al. 2016). In land plants, chromoplasts originate from pre-existing plastids like chloroplasts and store carotenoids in PG or bound to proteins (Li and Yuan 2013). This mechanism parallels β-carotene storage in *Dunaliella* species. Chloroplast budding during chromoplast formation, though rare, occurs in systems such as the *suffulta* tomato mutant (Forth and Pyke 2006). We propose that chloroplast-derived carotenoid–rich PG budding represents an ancient chromoplast biogenesis pathway, suggesting some land plants may have evolved new genes (e.g. *suffulta*) while losing this ancestral mechanism. We propose a model where stress-induced PG originating from thylakoid membranes bud toward the cytoplasm, enlarging before detaching (Fig. 2B). Released PG/CLD merge with TAG-rich CLD, forming astaxanthin-enriched lipid bodies. Chloroplast–CLD membrane interactions may also facilitate β-carotene transfer. Vacuolar budding provides an alternative transport route, collectively enabling β-carotene redistribution into astaxanthin-storing CLD.

In conclusion, this study provides insights into the transport of β-carotene in *H. pluvialis*, revealing the dynamic processes involved in PG budding, vesicle-mediated transport, and the formation of astaxanthin-rich lipid bodies. Future research focused on the functional characterization of the identified genes and the regulatory mechanisms controlling these pathways will be crucial to fully understanding carotenoid trafficking in *H. pluvialis*.

## Supplementary Material

kiaf423_Supplementary_Data

## Data Availability

Transcriptome data are available at NCBI Sequence Read Archive (https://www.ncbi.nlm.nih.gov/sra) with accession number PRJNA1242220. The data of supporting this study are available upon request from the corresponding author.

## Supplementary data

The following materials are available in the online version of this article.

**Supplementary Figure S1.** Astaxanthin content per dry weight in *H. pluvialis* under low-light (25 *µ*mol m^−2^ s^−1^) induction.

**Supplementary Figure S2.** Inhibition of carotenogenesis using 40 µm xanthohumol (DGAT inhibitor) and 40 µm DPA (BKT inhibitor) during 24 h induction.

**Supplementary Figure S3.** Effect of inhibitor DPA (40 µm) and Xanthohumol (40 µm) on the major carotenoid content in *H. pluvialis* NIES-144 under low-light induction conditions for 24 h.

**Supplementary Figure S4.** Isolation of chloroplasts from *H. pluvialis* under low-light induction conditions.

**Supplementary Figure S5.** Subcellular localization of astaxanthin in isolated chloroplasts.

**Supplementary Figure S6.** TEM observation of *H.*  *pluvialis* induced under low-light for 0–48 h.

**Supplementary Figure S7.** Other possible way of β-carotene transport in *H. pluvialis*.

**Supplementary Figure S8.** Differential expression genes with Go annotation of ESCRT, multivesicular, vacuolar transport, clathrin, vesicle, budding, or SNAR in *H.*  *pluvialis* treated under low-light inductions conditions for 72 h.

**Supplementary Figure S9.** Relative gene expression of the seven unique genes under high-light (200 µmol m^−2^ s^−1^) stress conditions.

**Supplementary Table S1.** In silico analysis DEGs related to vesicle transport or budding that are absent in *Dunaliella* sp.

**Supplementary Table S2.** Primer list.

**Supplementary Movie S1.**  *H. pluvialis* motility under low-light induction.

**Supplementary File S1.** DEGs with Go annotation of vesicle.
