# Supplementary material for: Chloroplast budding mediates β-carotene transport for early stage astaxanthin hyperaccumulation in microalgae
Source: Plant Physiol. 2025 Oct 13;199(2):kiaf423. doi: 10.1093/plphys/kiaf423 (PMC12516493; doi:10.1093/plphys/kiaf423)
Supplement: kiaf423_Supplementary_Data [file kiaf423_supplementary_data.zip › PLPHYS-2025-0652R2_Supplementary Movie Legend.pdf]

This movie depicts the motility of *H. pluvialis* cells under low-light induction (25 µmol m^{-2} s^{-1}). It is a composite of four short clips showing algal cell status at 0, 24, 48, and 72 h. Each original clip is approximately 3 seconds long and is shown at real speed (not accelerated). The total duration is 12 seconds. The footage was captured using a microscope with a 40x objective lens.
