# Supplementary material for: Chloroplast budding mediates β-carotene transport for early stage astaxanthin hyperaccumulation in microalgae
Source: Plant Physiol. 2025 Oct 13;199(2):kiaf423. doi: 10.1093/plphys/kiaf423 (PMC12516493; doi:10.1093/plphys/kiaf423)
Supplement: kiaf423_Supplementary_Data [file kiaf423_supplementary_data.zip › Supplementary Data.pdf]

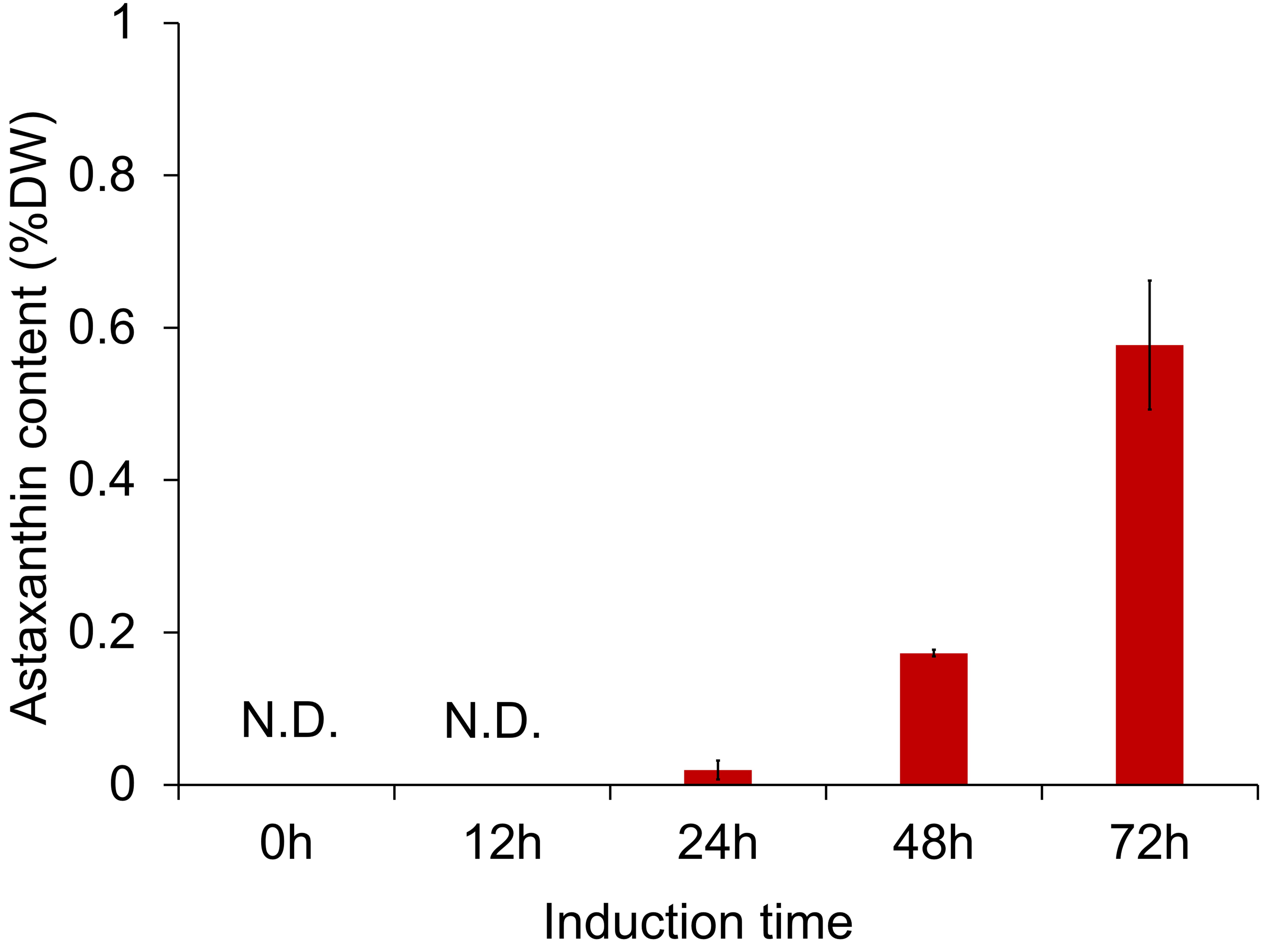

Supplementary Figure S1 Astaxanthin content per dry weight in *H. pluvialis* under low-light (25 μmol m^{-2} s^{-1}) induction. Data are expressed as mean ±SD (n=3 biological replicates). Pigments from algal cells were extracted and analyzed as described previously (Li et al., 2010; Ma et al., 2022). DW: dry weight. N.D.: not detected.

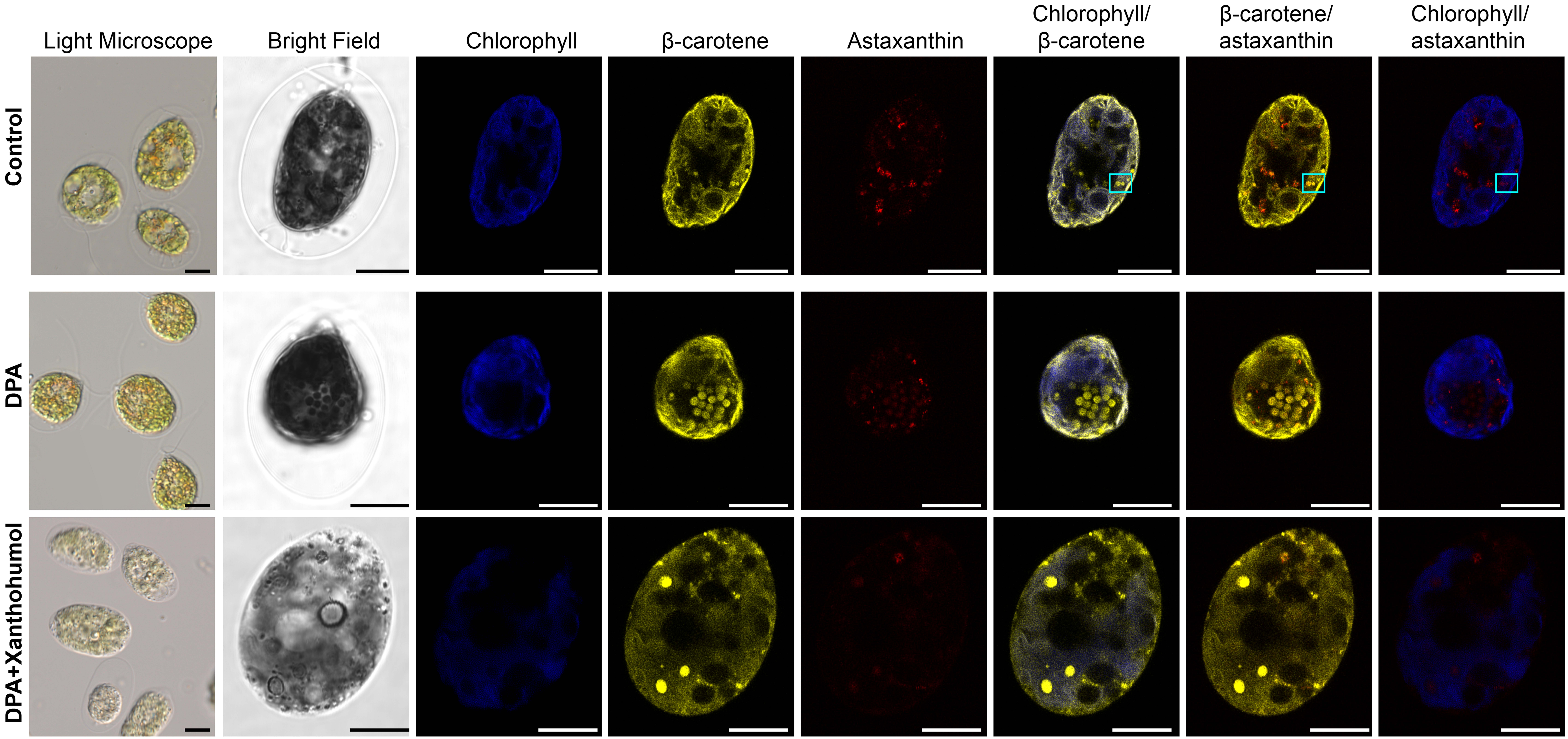

Supplementary Figure S2 Inhibition of carotenogenesis using 40 μM xanthohumol (DGAT inhibitor) and 40 μM diphenylamine (BKT inhibitor) during 24h induction. Parallel cultures at 2×10^5 cells mL^{-1} were divided into three experimental groups: (1) BKT inhibition: 40 μM diphenylamine (DPA); (2) Dual inhibition: 40 μM DPA + 40 μM xanthohumol; (3) Control: No inhibitors. All groups were exposed to continuous 25 μmol photons m^{-2} s^{-1} light at 22°C for 24 hours. Samples were analyzed exclusively by bright-field microscopy and confocal microscopy, Composite images show cellular morphology (brightfield) and pigment autofluorescence (Ota et al., 2018; Van Riel et al. 1983): Chlorophyll, λ_{ex}620 nm/λ_{em}700 nm (pseudocolored dark blue); β-carotene, λ_{ex}440 nm/λ_{em}520 nm (pseudocolored yellow); Astaxanthin, λ_{ex}540 nm/λ_{em}600 nm (pseudocolored red). The cyan boxes in the first panel suggest the co-localization of β-carotene, astaxanthin and chloroplast. Scale bar: 10 μm.

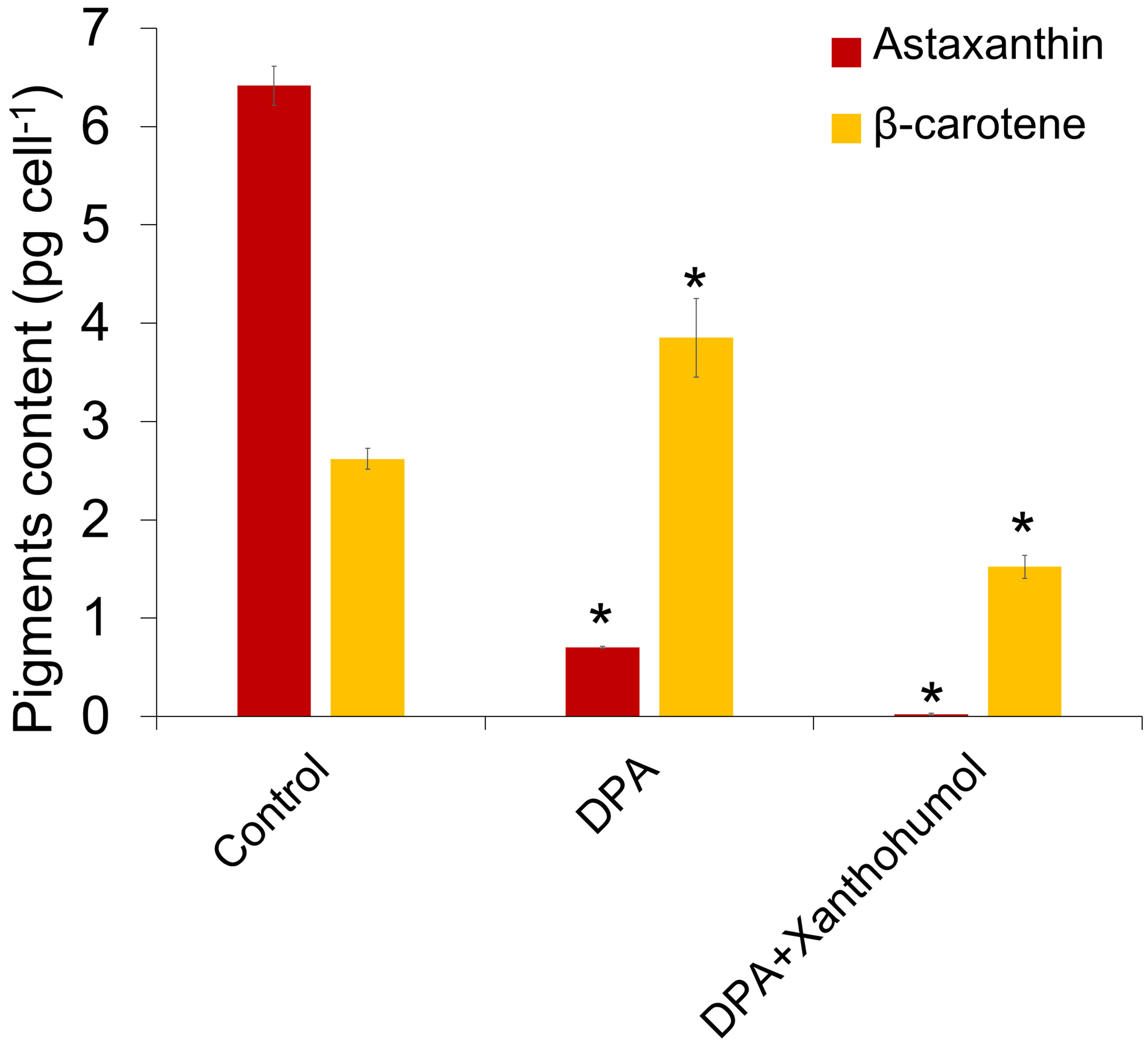

Supplementary Figure S3 Effect of inhibitor DPA (40 μM) and Xanthohumol (40 μM) on the major carotenoid content in *H. pluvialis* NIES-144 under low-light induction conditions for 24h. Pigments were analyzed by HPLC. Data are expressed as mean ±SD (n=3 biological replicates). *P<0.05 (Student's *t*-test). DPA: diphenylamine.

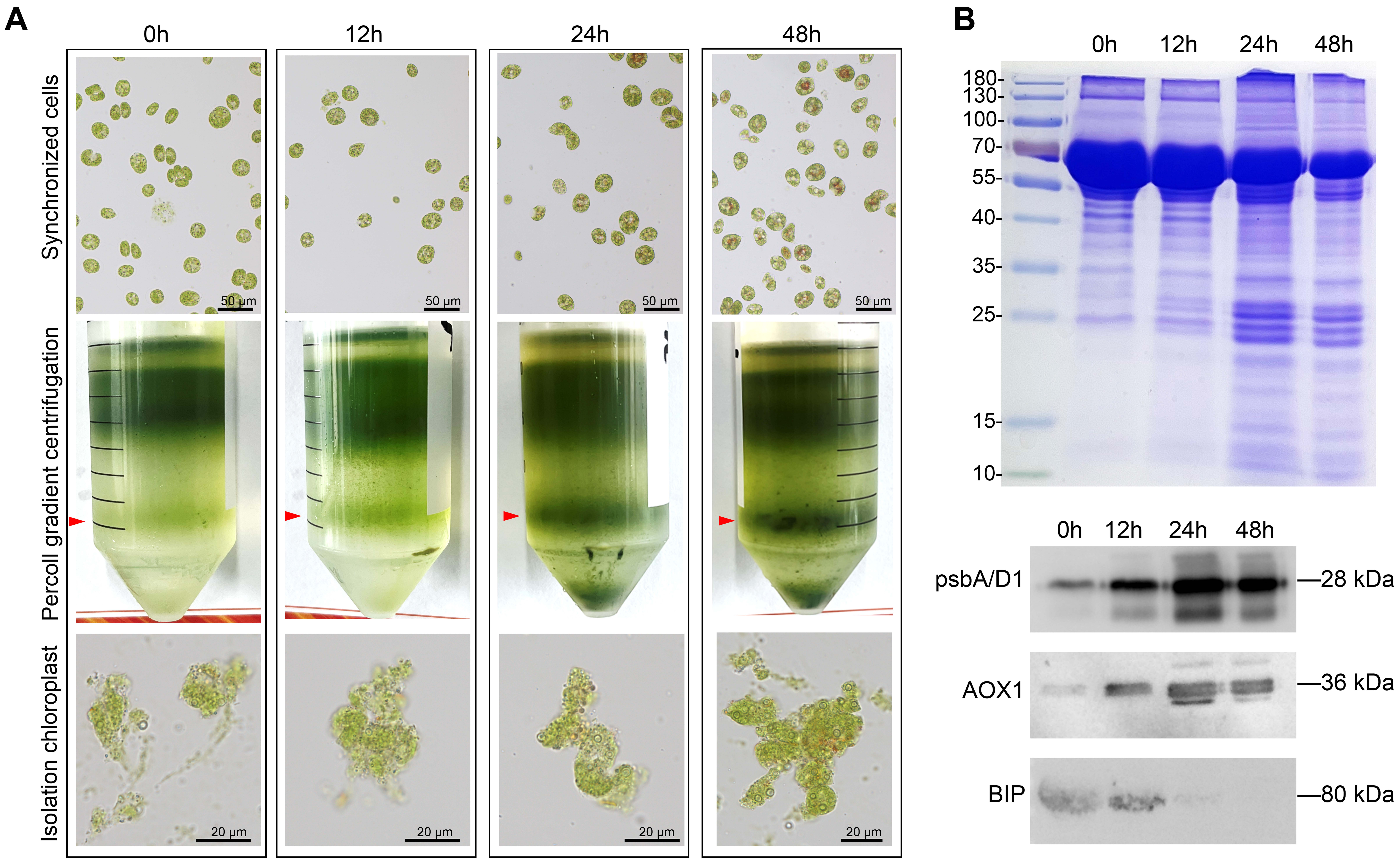

Supplementary Figure S4 Isolation of chloroplasts from *H. pluvialis* under low-light induction conditions. (A) The process of chloroplast isolation: Algal cells were harvested, washed once with phosphate-buffered saline (PBS), and resuspended in a hypertonic buffer (0.1 M Tris-HCl, pH 6.8; 0.3 M sorbitol) (Grunewald et al., 2001). After subjecting the cells to hypertonic shock, they were disrupted using a 10 μm pore-sized polycarbonate filtration membrane (Millipore). The resulting filtrate was centrifuged at 1500 g for 5 minutes at 4°C to separate chloroplasts from cell debris. The supernatant was discarded, and the pellet was resuspended in 2 mL of separation buffer (300 mM sorbitol, 50 mM HEPES-KOH, pH 7.5). This mixture was layered onto a Percoll gradient and centrifuged at 15,000 g for 15 minutes (Mason et al., 2006). Chloroplasts were located at the 45-65% interface of the gradient (as indicated by red arrowheads), while cell debris settled at the bottom. The chloroplast fraction was carefully collected, diluted with separation buffer, and subjected to low-speed centrifugation (750 g for 1 minute) to isolate the chloroplasts. (B) Purity check of chloroplast fractions isolated through western blot (Chen et al, 2015). The psbA/D1, AOX1 and BIP is the protein marker of chloroplast, mitochondria, and endoplasmic reticulum, respectively.

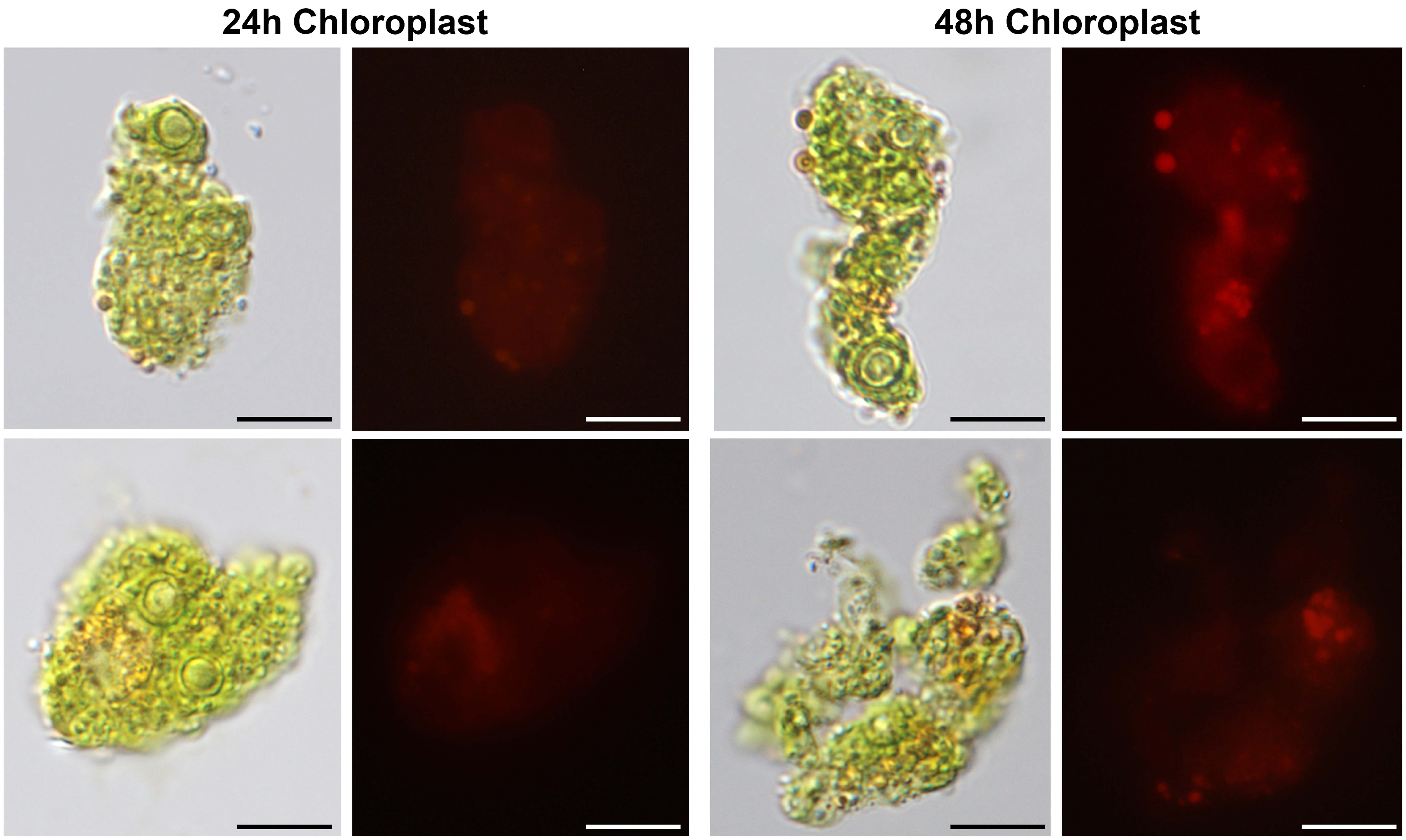

Supplementary Figure S5 Subcellular localization of astaxanthin in isolated chloroplasts. Fluorescent detection channel: Cy3. Scale bar: 10 μm.

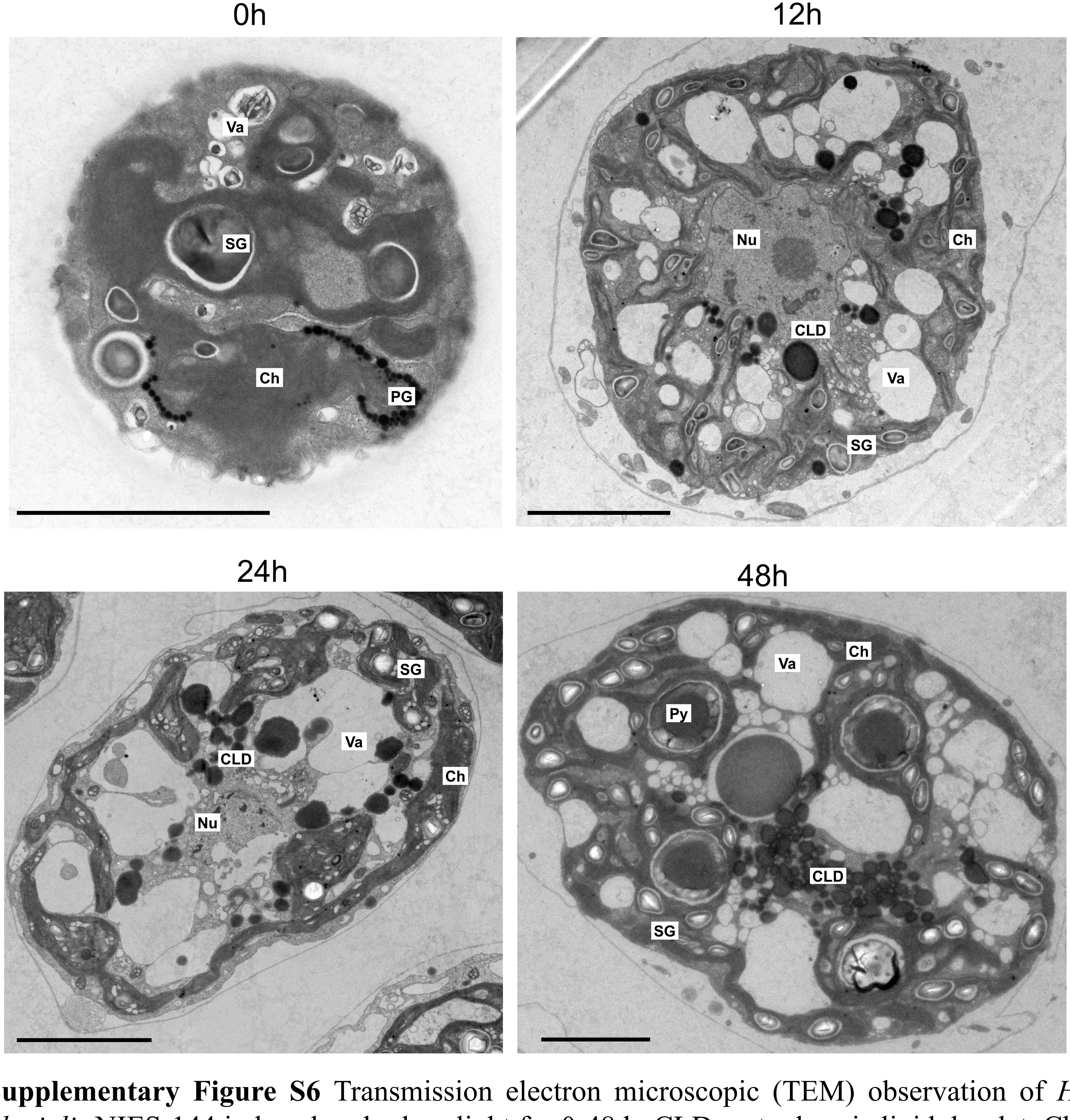

Supplementary Figure S6 Transmission electron microscopic (TEM) observation of *H. pluvialis* NIES-144 induced under low-light for 0-48 h. CLD: cytoplasmic lipid droplet. Ch: chloroplast. Va: vacuole. Nu: Nuleus. SG: Starch grain. Py: pyrenoid. Scale bar: 5 µm

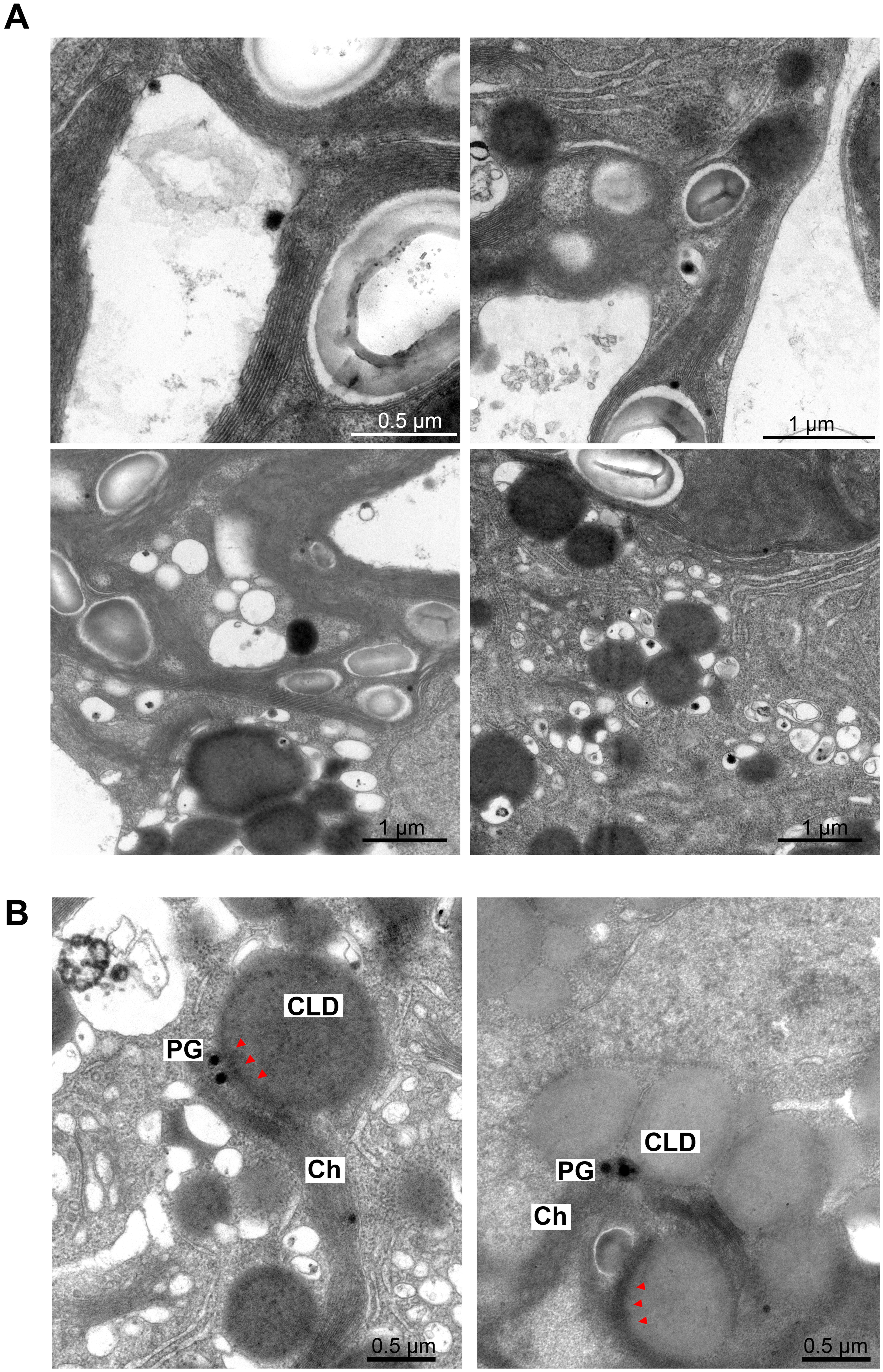

Supplementary Figure S7 Other possible way of β-carotene transport in *H. pluvialis*. (A) Vacuole-mediated PG trafficking pathway. (B) Membrane contact sites between CLDs and chloroplasts (red arrows) showing concentrated osmiophilic particles, indicative of active PG and CLD exchange. PG: plastoglobuli. CLD: cytoplasmic lipid droplet. Ch: chloroplast.

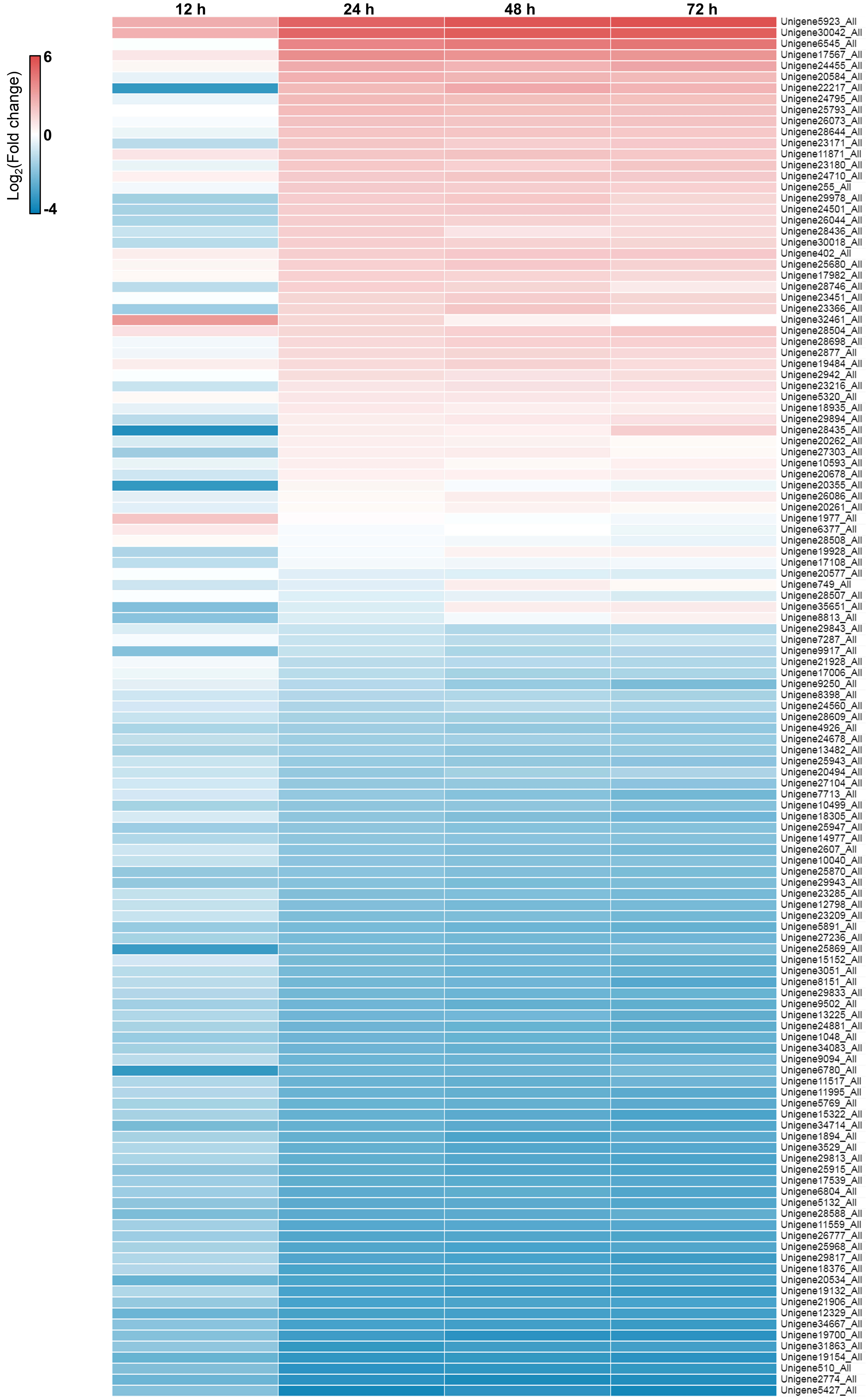

Supplementary Figure S8 Differential expression genes with Go annotation of ESCRT, multivesicular, vacuolar transport, clathrin, vesicle, budding, or SNAR in *H. pluvialis* treated under low-light inductions conditions for 72h.The heatmap illustrates the fold change, Log_2(Low-light induction for induced time/0h), of each gene with three biological replicates.

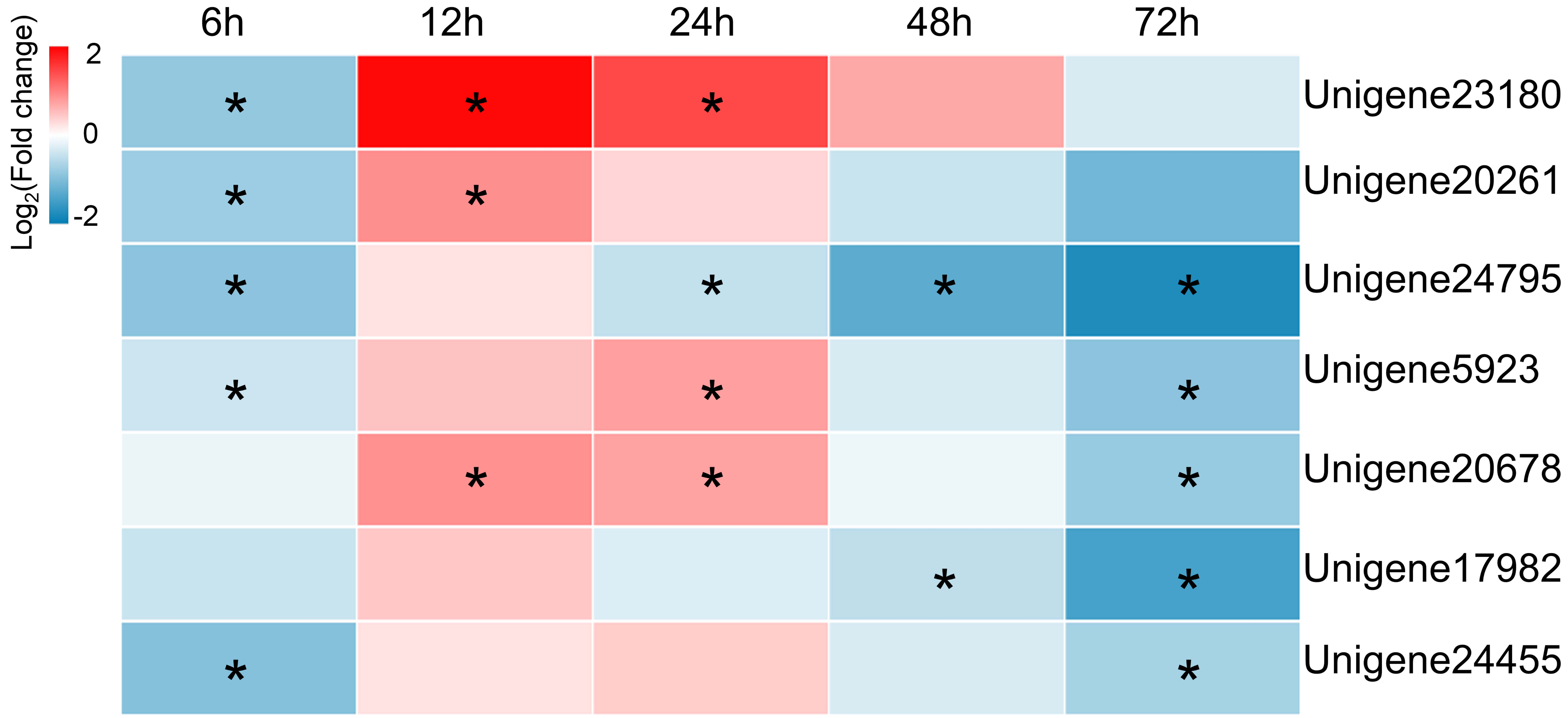

Supplementary Figure S9 Relative gene expression of seven unique genes under high-light (200 μmol m^{-2} s^{-1}) stress conditions. Values represent Log_2 fold change (high-light induction at indicated time points vs. 0h control) with three biological replicates. 18s rRNA served as the internal reference gene. Primers sequences are listed in Table S2. Gene expression was quantified by reverse transcription–quantitative PCR (RT-qPCR) techniques. *$P<0.05$ (Student's *t*-test). The relative fold change in expression was calculated as $2^{-\Delta\Delta Ct}$ (Livak and Schmittgen, 2001).

Supplementary References

Chen G, Wang B, Han D, Sommerfeld M, Lu Y, Chen F, Hu Q (2015) Molecular mechanisms of the coordination between astaxanthin and fatty acid biosynthesis in *Haematococcus pluvialis* (Chlorophyceae). Plant J **81:** 95-107

Grunewald K, Hirschberg J, Hagen C (2001) Ketocarotenoid biosynthesis outside of plastids in the unicellular green alga *Haematococcus pluvialis*. J Biol Chem **276:** 6023-6029

Li Y, Sommerfeld M, Chen F, Hu Q (2010) Effect of photon flux densities on regulation of carotenogenesis and cell viability of *Haematococcus pluvialis* (Chlorophyceae). J Appl Phycol **22:** 253-263

Livak KJ, Schmittgen TD (2001) Analysis of Relative Gene Expression Data Using Real-Time Quantitative PCR and the $2^{-\Delta\Delta CT}$ Method. Methods **25:** 402-408

Ma H, Zheng J, Li Y, Zhao L, Zou S, Hu Q, Han D (2022) A novel bifunctional wax ester synthase involved in early triacylglycerol accumulation in unicellular green microalga *Haematococcus pluvialis* under high light stress. Front Bioeng Biotech **9**

Mason CB, Bricker TM, Moroney JV (2006) A rapid method for chloroplast isolation from the green alga *Chlamydomonas reinhardtii*. Nat Protoc **1:** 2227-2230

Ota S, Morita A, Ohnuki S, Hirata A, Sekida S, Okuda K, Ohya Y (2018) Carotenoid dynamics and lipid droplet containing astaxanthin in response to light in the green alga *Haematococcus pluvialis*. Sci Rep **8**: 5617

Van Riel M, Hammans JK, Van De Ven M, Verwer W, Levine YK (1983) Fluorescence excitation profiles of beta-carotene in solution and in lipid/water mixtures. Biochem Biophys Res Commun **113**: 102-107

Supplementary Table S1 *In silico* analysis DEGs related to vesicle transport or budding that are absent in *Dunaliella* sp.

ID	GO Term	Homologs	Annotation	Conserved domains	Predicted localization
Unigene23180_All	[P]GO:0016192//vesicle-mediated transport	No	No	No	*Cytoplasm, nucleus, cell membrane, or mitochondrion \Soluble \ None
Unigene20261_All	[P]GO:0007015//actin filament organization; GO:0030050//vesicle transport along actin filament; [C]GO:0015629//actin cytoskeleton; GO:0016459//myosin complex; GO:0031982//vesicle; GO:0005737//cytoplasm; [F]GO:0003774//cytoskeletal motor activity; GO:0051015//actin filament binding; GO:0005516//calmodulin binding; GO:0005524//ATP binding; GO:0000146//microfilament motor activity; GO:0003779//actin binding	*C. Reinhardtii* (22% coverage)	MYO3	No	**Not available
Unigene24795_All	[P]GO:0006886//intracellular protein transport; GO:0090522//vesicle tethering involved in exocytosis	*C. reinhardtii* (90% coverage)	hypothetical protein	No	Lysosome/vacuole\ Peripheral, soluble \Signal peptide
Unigene5923_All	[P]GO:0015031//protein transport; GO:0006891//intra-Golgi vesicle-mediated transport; [C]GO:0017119//Golgi transport	*C. Reinhardtii* (99% coverage)	Esterase, Alpha/Beta hydrolase	YbbA, predicted hydrolase of the alpha/beta	Cytoplasm\ Soluble\ None

	complex; GO:0000139//Golgi membrane; [F]GO:2001070//starch binding			superfamily; Esterase	
Unigene20678_All	[P]GO:0016192//vesicle-mediated transport; GO:0006886//intracellular protein transport; GO:0048280//vesicle fusion with Golgi apparatus; [C]GO:0000139//Golgi membrane; GO:0005794//Golgi apparatus	All but *Dunaliella*	Golgin candidate 6-like	No	Cytoplasm\ Peripheral, soluble\ None
Unigene17982_All	[P]GO:0042147//retrograde transport, endosome to Golgi; GO:0032456//endocytic recycling; [C]GO:0005829//cytosol; GO:1990745//EARP complex; [F]GO:0000149//SNARE binding;	All but *Dunaliella*	Syndetin	Vacuolar-sorting protein 54; DUF2451	Cytoplasm\ Peripheral, soluble\ None
Unigene24455_All	[P]GO:0006887//exocytosis; GO:0006893//Golgi to plasma membrane transport; GO:0090522//vesicle tethering involved in exocytosis; GO:0006904//vesicle docking involved in exocytosis; GO:0006612//protein targeting to membrane; [C]GO:0000145//exocyst	All but *Dunaliella, and Tagetes erecta* L.	Sec8_exocyst domain-containing protein	Sec8_exocyst: Sec8 excocyst complex component specific domain	**Not available

Protein subcellular localization was predicted using DeepLoc-2.1 (https://services.healthtech.dtu.dk/services/DeepLoc-2.1/). DeepLoc 2.1 distinguishes among 10 distinct subcellular localizations and 4 membrane associations states. The localization categories include: Nucleus, Cytoplasm, Extracellular, Mitochondrion, Cell membrane, Endoplasmic reticulum, Chloroplast, Golgi apparatus, Lysosome/Vacuole and Peroxisome. Membrane association is classified into four types: Peripheral, Transmembrane, Lipid anchor and Soluble (non-membrane). Additionally, DeepLoc 2.1 predicts potential sorting signal(s) that influence the subcellular localization(s). *For Unigene23180, the predicted localization scores fell the confidence threshold; therefore, the top four most probable localizations are reported. **No localization prediction was available due to the absence of an initial codon in the predicted CDS sequence.

Supplementary Table S2 Primer list

Name	Sequence (5'-3')
Unigene23180_qF	GTGACCATCACACAGGGGAG
Unigene23180_qR	AAAGCCTTGACCCTAACCCG
Unigene20261_qF	ACATGTGGCATAGGGAGTACG
Unigene20261_qR	ACTGAACAGCTTGCCCATCA
Unigene24795_qF	AACTGCTACAGGAGGGCAAC
Unigene24795_qR	CTGACATGCCATCCTCCACG
Unigene5923_qF	GTACTGCCAGGGTTTGTGGT
Unigene5923_qR	CACGCGATACGTTTGCTGAG
Unigene20678_qF	CAGTCGATTCTTGGCAGGGT
Unigene20678_qR	CAGCTCCTTCAGCTGGTTCA
Unigene17982_qF	ACCTTCATGGTGCTGTCAGG
Unigene17982_qR	TGCCTTCTGCTGTGAGGATG
Unigene24455_qF	CGAGCAAGTGGACTGGGAAT
Unigene24455_qR	TCCACCACCATGTCAACGAG
18s rRNA-qF	TGCCTAGTAAGCGCGAGTCA
18s rRNA-qR	CCCACCGCTAAAGTCAATCC
